# Severe Rhabdomyolysis due to Presumed Drug Interactions between Atorvastatin with Amlodipine and Ticagrelor

**DOI:** 10.1155/2017/3801819

**Published:** 2017-05-25

**Authors:** Iouri Banakh, Kavi Haji, Ross Kung, Sachin Gupta, Ravindranath Tiruvoipati

**Affiliations:** ^1^Department of Pharmacy, Frankston Hospital, Peninsula Health, Frankston, VIC 3199, Australia; ^2^Department of Intensive Care Medicine, Frankston Hospital, Peninsula Health, Frankston, VIC 3199, Australia; ^3^School of Public Health, Faculty of Medicine, Nursing and Health Sciences, Monash University, Clayton, VIC 3800, Australia

## Abstract

Atorvastatin and ticagrelor combination is a widely accepted therapy for secondary prevention of ischaemic heart disease. However, rhabdomyolysis is a well-known rare side effect of statins which should be considered when treatments are combined with cytochrome P450 3A4 enzyme inhibitors. We report a case of atorvastatin and ticagrelor associated severe rhabdomyolysis that progressed to multiorgan failure requiring renal replacement therapy, inotropes, intubation, and mechanical ventilation. Despite withdrawal of the precipitating cause and the supportive measures including renal replacement therapy, creatinine kinase increased due to ongoing rhabdomyolysis rapidly progressing to upper and lower limbs weakness. A muscle biopsy was performed to exclude myositis which confirmed extensive myonecrosis, consistent with statin associated rhabdomyolysis. After a prolonged ventilatory course in the intensive care unit, patient's condition improved with recovery from renal and liver dysfunction. The patient slowly regained her upper and lower limb function; she was successfully weaned off the ventilator and was discharged for rehabilitation. To our knowledge, this is a second case of statin associated rhabdomyolysis due to interaction between atorvastatin and ticagrelor. However, our case differed in that the patient was also on amlodipine, which is considered to be a weak cytochrome P450 3A4 inhibitor and may have further potentiated myotoxicity.

## 1. Introduction

Statins are a widely used class of drugs that has an established benefit in patients with ischaemic heart disease (IHD) at the highest tolerated doses [1–3]. Statin associated rhabdomyolysis (SAR), although rare, is a well-recognized life threatening adverse effect [4]. “Rhabdomyolysis is a severe form of muscle damage associated with very high creatinine kinase (CK) levels, with myoglobinaemia and/or myoglobinuria with a concomitantly increased risk of renal failure” [4]. The rise of CK during rhabdomyolysis that is associated with lipid lowering therapy is usually more than 10 times upper limit of normal [5]. The risk of SAR is increased with increased statin potency, increased statin blood concentration, age greater than 75 years, female gender, and low body mass index [4]. This is potentiated by patient characteristics, preexisting comorbidities such as hepatic, renal, metabolic, or neuromuscular diseases, and drug interactions [4].

The incidence of SAR is rare, estimated at 1 per 100,000 per year, but the risk may be increased when statins are combined with Cytochrome P450 3A4 (CYP3A4) enzyme inhibitors [4]. Here we present a case report of an elderly patient with a diagnosis of SAR due to presumed cardiovascular drug interactions with several intrinsic factors for the adverse event.

## 2. Case Presentation

A 74-year-old Maltese female was transferred to our hospital from a rural emergency department following an unwitnessed collapse preceded by several days of generalized weakness. Her significant past medical history included ST elevated myocardial infarction, hypertension, depression, osteoarthritis requiring a total hip replacement, and osteoporosis. Her weight was stable at 51 kg with a body mass index of 22.5. She was a nonsmoker and she consumed on average one unit of alcohol per day. Her admission medications included amlodipine, atorvastatin, ticagrelor, metoprolol, aspirin, amitriptyline, perindopril, and weekly risedronate. She had been treated with a combination product of amlodipine and atorvastatin for several years. Two and a half months prior to her admission, she was diagnosed with ST elevation myocardial infarction, which was medically managed due to unsuccessful percutaneous coronary intervention to reopen a blocked artery. Her management included an increased dose of amlodipine/atorvastatin combination from 5/20 mg to 5/80 mg and antiplatelet therapy of low-dose aspirin in addition to ticagrelor 90 mg twice a day as per treatment guidelines.

In the rural emergency department, the patient was hypotensive and had minimum urine output. She received fluid resuscitation of 4 litres and was commenced on noradrenaline infusion at 10 micrograms per minute. The initial diagnosis was septic shock and acute kidney injury with a creatinine level of 404 *μ*mol/L and urea of 17 mmol/L. She had mild neutrophilia. The chest X-ray and computed tomography of the brain and the cervical spine were reported as unremarkable. She was then transferred to our intensive care unit (ICU) due to lack of ICU services at the referring hospital.

Upon admission to ICU, the patient appeared confused, but cooperative. She was moving her 4 limbs. Her heart rate, blood pressure, respiratory rate, and temperature were 86 beats per minute, 102/42 mmHg, 15 breaths per minute, and 35.7°C, respectively, while receiving 10 micrograms per minute of noradrenaline. She was well oxygenated on 2 litres per minute of oxygen. She was tender on her right lumber region, while the rest of the physical examination was unremarkable. The liver function was significantly deranged, with alteration in the coagulation profile and worsening renal function (Table 1).

The computed tomography and the ultrasound of the abdomen revealed a calculus thickened gall bladder with pericholecystic fluid and free fluid in the abdomen. The diagnosis of acute cholecystitis that resulted in multiorgan failure was affirmed. On subsequent assessment however, the abdominal symptoms and signs had dissipated, and surgery was no longer indicated. Continuous venovenous haemodialysis and filtration (CVVHDF) was commenced due to worsening metabolic acidosis and acute anuric renal failure.

**Figure 1:**
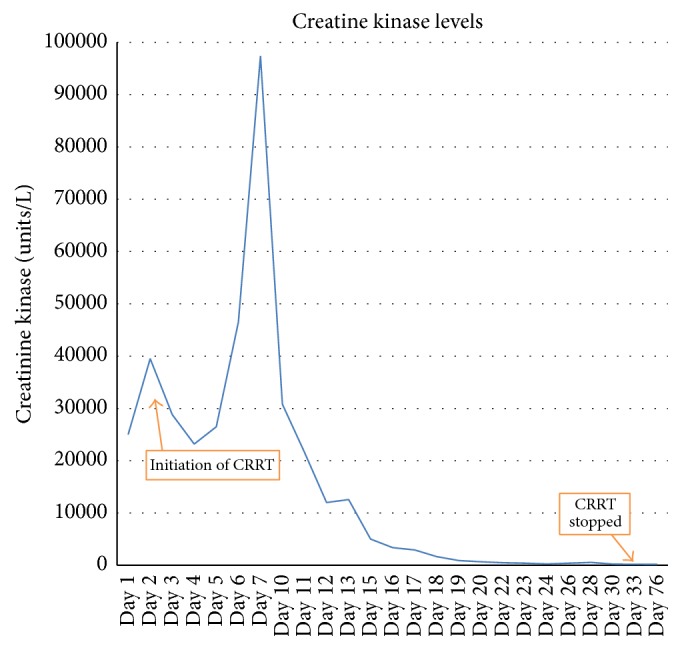
Changes in creatinine kinase levels during the course of patient stay.

On day 2, the patient developed worsening muscle pain and progressive weakness in the upper and lower limbs with diminished tendon reflexes. The creatinine kinase (CK) was profoundly elevated approaching 100000 U/L (Figure 1) and there was a progressive deterioration in the liver function (Table 1). On further microbiological, biochemical, and serological assessment, sepsis, haemolysis, vasculitis, thyroid disorders, and paracetamol toxicity were excluded. A provisional diagnosis of autoimmune myositis or SAR was suggested. On day 7 magnetic resonance imaging (MRI) of the musculoskeletal system revealed features consistent with upper and lower limbs proximal myositis. MRI targeted muscle biopsy was also performed. CK was persistently elevated with worsening liver function; disseminated intravascular coagulation developed leading to epistaxis, upper gastrointestinal bleeding, and subcutaneous haemorrhage. She continued to have moderate neutrophilia, modest increase in the C-reactive protein.

The disseminated intravascular coagulopathy was treated with platelets and clotting factors replacement and with Vitamin K. A presumed diagnosis of autoimmune myositis was made. Further deterioration in the patient state, while waiting for the biopsy results, prompted treatment with 1 gram of intravenous methylprednisolone daily for 3 consecutive days for the potential autoimmune aetiology of her presentation. The patient was more fatigued, becoming drowsy and hypoxic and requiring endotracheal intubation and ventilation. The muscle biopsy revealed extensive myonecrosis of similar age and scant regeneration, consistent with toxin induced necrotising myositis.

Her condition gradually improved and CK levels gradually returned to within the normal range (Figure 1). Due to profound weakness, she was rendered difficult to be weaned off mechanical ventilation and subsequently received percutaneous tracheostomy. The renal and liver function gradually improved and CVVHDF was discontinued. The renal biopsy confirmed acute tubular necrosis with extensive myoglobin-related cast formation. She was gradually weaned off the ventilator and the tracheostomy tube was removed in the ICU. She was mechanically ventilated for a total of 1041 hours and she stayed in ICU for 69 days. She was discharged to the medical ward and subsequently discharged to a rehabilitation centre.

## 3. Discussion

Our case highlights a serious drug induced myotoxicity from high dose atorvastatin, two and a half months after instituting ticagrelor and continuing with amlodipine. A coprescribed statin and a P2Y12 inhibitor are standard therapy for secondary prevention of IHD. However, possibilities of drug interactions in this patient were overlooked. To our knowledge this is the second case of SAR due to an interaction between atorvastatin and ticagrelor [6]. Kido et al. [6] reported a case of similar interaction that led to rhabdomyolysis as the adverse event, though their patient was over a decade younger and was obese. Notably, our patient was also receiving amlodipine, a calcium channel blocker, which may have further contributed to SAR [7]. The patient presented in this case report had no other known causes or predisposing factors for rhabdomyolysis such as trauma, ischaemia, or metabolic disorders [5]. However, rarer causes of rhabdomyolysis such as mitochondrial disorders or inherited gene mutations were not investigated as the there was a clear drug-related precipitating factor identified early into patient's presentation, as well as the muscle biopsy supporting the diagnosis with a picture consistent with toxin related muscle necrosis [5].

Ticagrelor is an oral antiplatelet agent that binds reversibly to P2Y12 receptors. Ticagrelor differs from clopidogrel, in that it has a faster onset of action, higher platelet inhibition, and a higher morbidity reduction [8]. Dose adjustment is not required to prevent bleeding excluding severe hepatic impairment irrespective of age, gender, ethnicity, or renal impairment [9]. There is a minor increase in the myotoxic effect of atorvastatin when combined with ticagrelor, with area under concentration (AUC) curve 1.4-fold increase, while simvastatin AUC is increased 2-3-fold [10]. Ticagrelor is a known weak inhibitor of the CYP3A enzymes and P-glycoprotein, with the risk of drug interactions with other drugs affected by these pathways considered to be low [9, 11]. The latest American Heart Association (AHA) recommendation from 2016 for using the atorvastatin and ticagrelor is that the “combination is reasonable” [10]. While others have suggested that the interaction between ticagrelor and statins metabolised via CYP3A4 may have provided an added benefit in the primary ticagrelor registration study [12], the interaction may have clinical implications from a safety point of view also [6]. Coadministration of ticagrelor with a high dose of atorvastatin 80 mg had led to a clinically significant rise in statin concentration in our patient's case. Although this is considered a minor risk in young healthy volunteers [9], such a combination with a large dose of atorvastatin was sufficient to cause extensive rhabdomyolysis in our patient. Further, the latest guideline from the AHA has classified ticagrelor as low risk drug interaction for myotoxicity when coadministered with atorvastatin and has recommended that no dose alteration is required [10].

Our patient was also exposed to another drug that may have contributed to the increased risk of rhabdomyolysis. Amlodipine is a dihydropyridine calcium channel blocker that is frequently used for hypertension and angina management [13]. It is frequently combined and sold as a combination product with atorvastatin (Caduet®). In healthy volunteers this product has been found to be safe. One study showed no change in the maximum concentration of atorvastatin from the combination, with the AUC for atorvastatin increasing by 18% [13]. Amlodipine is a weak inhibitor of both CYP3A4 and CYP3A5 enzymes [13, 14] and may effectively not only contribute to a higher atorvastatin AUC, but also increase exposure to ticagrelor and its active metabolite. This boost to ticagrelor levels may in turn add to higher atorvastatin AUC contribution due to a greater extent of enzyme inhibition.

## 4. Conclusion

This case illustrates that when high doses of atorvastatin are coadministered with ticagrelor, as secondary prevention for IHD, the combination may pose a risk for serious myotoxicity. Moreover, multiple therapies with CYP3A inhibitors, which in this case were ticagrelor and amlodipine, may further increase the risk of statin associated rhabdomyolysis. Further, awareness of statin myotoxic risks should always be raised in susceptible patients; close monitoring and dose alteration are recommended when multiple CYP3A inhibitors are administered.

## Figures and Tables

**Table 1:** Changes in haematological and biochemical parameters during the course of the disease.

Parameter	Day 1	Day 6	Day 7	Day 8	Day 11	Day 13	Day 69
Haemoglobin (115–165 g/L)	119	77	104	82	95	88	91
Platelets (150–450)	251	113	170	136	23	29	404
INR (<1.3)	1.8	1.7	1.7	2	1.8	1.8	1.3
APTT (26–36 seconds)	53	41	39	49	40	107	28
Urea (3–10 mmol/L)	18.4	8.7	12.8	11.2	12	9.1	11
Creatinine (40–80 micromol/L)	480	143	173	93	75	48	88
Estimated GFR (>60 mL/min/1.73 m^2^)	7	31	25	52	68	90	55
Total bilirubin (<15 micromol/L)	53	108	157	156	167	163	10
ALT (0–30 units/L)	746	1094	1605	1537	1228	1346	30
AST (<35 units/L)	1153	1736	2591	2020	1219	890	46
GGT (<35 units/L)	527	348	399	387	234	224	134
ALP (30–115 units/L)	260	232	293	325	248	208	104
pH (7.38–7.43)	7.34	7.46	7.46	7.5	7.42	7.5	7.43
Bicarbonate (20–24 mmol/L)	11	23	15	21	22	24	34
Base excess (−3.3–1.2 mmol/L)	13.2	0.3	7.2	1.1	1.2	1.8	8.6
Lactate (0.5–2.0 mmol/L)	1.5	3.3	7.4	4.2	4.1	2.9	1.4

APTT: activated partial thromboplastin time, INR: International Normalised Ratio, and GFR: Glomerular Filtration Rate.
